# Mercury levels of marine fish commonly consumed in Peninsular Malaysia

**DOI:** 10.1007/s11356-014-3538-8

**Published:** 2014-09-26

**Authors:** Nurul Izzah Ahmad, Mohd Fairulnizal Mohd Noh, Wan Rozita Wan Mahiyuddin, Hamdan Jaafar, Ismail Ishak, Wan Nurul Farah Wan Azmi, Yuvaneswary Veloo, Mohd Hairulhisam Hairi

**Affiliations:** 1 Institute for Medical Research, Jalan Pahang, 50588 Kuala Lumpur, Malaysia; 2 Fisheries Biosecurity Centre, Department of Fisheries Malaysia, Lot 82, Jalan Carruthers, Off Jalan Sultan Salahuddin, 50480 Kuala Lumpur, Malaysia; 3 Fisheries Research Institute, 11960 Batu Maung, Penang Malaysia

**Keywords:** Total mercury, Marine fish, Demersal fish, Pelagic fish, Fish landing ports, Wet market, Peninsular Malaysia

## Abstract

This study was conducted to determine the concentration of total mercury in the edible portion of 46 species of marine fish (*n* = 297) collected from selected major fish landing ports and wholesale markets throughout Peninsular Malaysia. Samples were collected in June to December 2009. Prior to analysis, the fish samples were processed which consisted of drying at 65 °C until a constant weight was attained; then, it was grounded and digested by a microwave digestion system. The analytical determination was carried out by using a mercury analysis system. Total mercury concentration among fish species was examined. The results showed that mercury concentrations were found significantly higher (*p* < 0.001) in demersal fish (the range was from 0.173 to 2.537 mg/kg in dried weight) compared to pelagic fish (which ranged from 0.055 to 2.137 mg/kg in dried weight). The mercury concentrations were also higher in carnivorous fish especially in the species with more predatory feeding habits. Besides, the family group of *Latidae* (0.537 ± 0.267 mg/kg in dried weight), *Dasyatidae* (0.492 ± 0.740 mg/kg in dried weight), and *Lutjanidae* (0.465 ± 0.566 mg/kg in dried weight) showed significantly (*p* < 0.001) higher mercury levels compared to other groups. Fish collected from Port Klang (0.563 ± 0.509 mg/kg in dry weight), Kuala Besar (0.521 ± 0.415 mg/kg in dry weight), and Pandan (0.380 ± 0.481 mg/kg in dry weight) were significantly higher (*p* = 0.014) in mercury concentrations when compared to fish from other sampling locations. Total mercury levels were significantly higher (*p* < 0.002) in bigger fish (body length >20 cm) and were positively related with fish size (length and weight) in all fish samples. Despite the results, the level of mercury in marine fish did not exceed the permitted levels of Malaysian and JECFA guideline values at 0.5 mg/kg methylmercury in fish.

## Introduction

Fish is an important source of protein in Malaysia. Daily consumption of fish is on average one and a half medium fish per day (Norimah et al. 2008). The Malaysian per capita consumption of fish was 56.39 kg/person/year in the year 2003 and accounted for 12.4 % of total food intake per capita (Tey et al. 2008). The results of the Household Expenditure Survey for Malaysia in 2004/2005 showed that budget shares on fish (22.1 %) was the second largest after cereals (23.9 %), and the trend showed an increase when compared to the previous 1999/2000 survey (21.8 %). A study on food consumption behavior among Malays showed that the consumers were not affected by the changes in fish price, where an increase in the expenditure on fish may be caused by increment of income together with an increase in the health consciousness (Tey et al. 2008).

Many researchers have discussed the benefits of seafood and bioactive components from aquatic sources in relation to various health outcomes (Larsen et al. 2011; McManus et al. 2011; Torpy 2006). Marine fish has a favorable fatty acid composition, namely, the long-chain n-3 polyunsaturated fatty acids (n-3 PUFA), the eicosapentaenoic acid (EPA; C20:5n-3), and the docosahexaenoic acid (DHA; C22:6n-3) that has been linked to a lower incidence of cardiovascular disease (CVD) (Larsen et al. 2011; McManus et al. 2011; Torpy 2006). High-quality fish proteins contain all the essential amino acids and are highly digestible (Larsen et al. 2011). The bioactive properties from fish proteins and peptides have been reported to be an antihypertensive, antioxidative, anticoagulant, and antimicrobial components in functional foods or nutraceuticals and pharmaceuticals. Marine foods are also excellent source of essential nutrients such as minerals (iodine and selenium) and vitamins (vitamins A, D, and B_12_). Other marine bioactive components linked to health promoting effects include taurine, phytosterols, antioxidants, and phospholipids (Larsen et al. 2011; McManus et al. 2011; Torpy 2006).

Conversely, fish consumption is the major route of mercury exposure to human and it is often found in the form of methylmercury (Burger 2009; Morgano et al. 2011; Castro-Gonžalez and Mendez-Armenta 2008; Myers and Davidson 2000). Fish may concentrate methylmercury either directly through the water or through components of the food chain (Castro-Gonžalez and Mendez-Armenta 2008). Mercury attached to aquatic sediments is subject to microbial conversion to methylmercury, at which point it enters the aquatic food chain and reaches its highest concentration in predatory fish (Clarkson et al. 2003). The cyclic order of mercury contamination chain starts from its emission in industries. This is followed by contamination in atmosphere, soil, water, phytoplankton, zooplankton, fish, and to human (Castro-Gonžalez and Mendez-Armenta 2008). There are two important sources of mercury which are anthropogenic and natural sources. The most important source is from the anthropogenic sources particularly from urban discharges, agricultural materials, mining and combustion, and industrial discharges (Castro-Gonžalez and Mendez-Armenta 2008; Streets et al. 2005). Volcanic eruptions are believed to be an important natural source of mercury (Clarkson and Magos 2006).

Methylmercury is a robust toxicant, and the primary target is the central nervous system (Clarkson and Magos 2006) especially the brain tissue (Clarkson et al. 2003). Methylmercury is highly mobile in the human body where its passage across the blood-brain and placental barriers, cause damage, both prenatally and postnatally (Tollefson and Cordle 1986). It appears to be most neurotoxic prenatally when the brain is developing rapidly (Myers and Davidson 2000). The journey of methylmercury into the human body is explained through the formation of water-soluble methylmercury complexes in body tissues that are attached to thiol groups in protein, certain peptides, and amino acids (Clarkson and Magos 2006). It may enter into body cells as methylmercury-cysteine complex and exit via glutathione pathway. The main route of its elimination from the body is via feces, which is as much as 90 % of total excretion according to animal observation (Clarkson and Magos 2006). In adults, the main symptoms of methylmercury exposure related with intoxication are to the nervous system, with paraestesia or numbness in the hands and feet, coordination difficulties, and concentric constriction of the visual field, auditory symptoms, ischemic stroke, dementia and depression. It might also cause nephrotoxicity and gastrointestinal toxicity with ulceration and hemorrhage (Castro-Gonžalez and Mendez-Armenta 2008; Clarkson and Magos 2006; Tollefson and Cordle 1986).

Scientists worldwide have researched the toxicity of methylmercury since the first outbreak which was reported in Minamata, Japan, in 1956. Numerous reports and review articles discussing these issues were published. Among the earliest publications, as has been cited by Myers et al. (2000), were publications by the researchers from the University of Rochester on measurement of exposure and the consequences of exposure to various forms of mercury on experimental animals. Later, Swedish investigators discovered the methylation process of inorganic mercury by organisms in the aquatic sediments and in fish which may concentrate methylmercury either directly through the water or through components of the food chain (Myers et al. 2000). Recent scientific publications have focused on the levels of methylmercury contamination in seafood, namely, fish (Denton et al. 2006; Guerin et al. 2011; Ikem and Egiebor 2005; Mendil et al. 2010; Turkmen et al. 2005; Yilmaz et al. 2010), human exposure, and its related health effects (Burger 2009; Morgano et al. 2011; Castro-Gonžalez and Mendez-Armenta 2008; Myers and Davidson 2000).

There are few publications on the concentrations of mercury in marine fish reported by Malaysian researchers. Most studies reported on various levels of mercury in limited marine fish species that collected from selected sites in Peninsular Malaysia only. Among the most recent research studies were carried out by Alina et al. (2012), Mok et al. (2012), Kamaruzaman et al. (2011), Hajeb et al. (2009, 2010), Irwandi and Farida (2009), and Agusa et al. (2005a, 2007). Most of the results from these research studies showed that mercury were found to have lower concentrations in marine fish compared to the permissible limits set either by Malaysian Standards or JECFA guideline values. Only Agusa et al. (2005a) reported that some species of the marine fish captured from Malaysian markets had high mercury concentrations that may cause hazardous to the consumers. Similarly, Hajeb et al (2009, 2010) reported the highest level of mercury in longtail tuna and short-bodied mackerel captured from both east and west coast of Peninsular Malaysia. In another separated study, their findings showed high mercury intake by fishermen families at the rural areas compared to the general adult population (Hajeb et al 2011).

Studies from several neighboring areas/countries such as Gresik Coast, Indonesia (Soegianto et al. 2010), Mekong Delta, South Vietnam (Ikemoto et al. 2008), East Coast of Thailand (Cheevaporn et al 2000), Mekong River, and several places in Cambodia (Murphy et al. 2008; Agusa et al. 2005b) showed that variability of mercury concentrations was quite high from species to species of marine fish. Murphy et al. (2008) reported mercury levels of some fish species of up to sixfold higher compared to the average of 99 ng/g mercury in Kratie Mekong, Cambodia. Some marine fish species such as thresher shark, tille travelly, skipjack tuna, swordfish (Sompongchaiyakul et al. 2011), sharp-tooth snapper, and obtuse barracuda (Agusa et al. 2005b) had mercury concentrations exceeded the JECFA guidelines. All these research studies reported on few limited species of marine fish in selected locations for their studies only. Therefore, information on level of mercury in various marine fish consumed is timely. The objectives of this study were aimed at determining and interpreting the concentrations of total mercury in the edible tissues of 297 commonly consumed marine fish samples that composed of 46 species, collected in June to December 2009 from fish landing ports and wholesale markets throughout Peninsular Malaysia. This study will provide baseline data of mercury levels in muscle of 12 species of *Carangidae*, 11 species of *Scrombidae*, 5 species of *Lutjanidae*, 2 species of *Latidae*, 4 species of *Dasyatidae*, 4 species of *Sciaenidae*, and 8 species of *Nemipteridae*. The relationship between mercury levels and fish size (length and weight) was investigated, and mercury burden sampled from fish at different habitats, family group, and areas were also compared. It is hoped that later, the data reported will serve as an invaluable baseline study for estimating and assessing risk on mercury contamination through seafood consumption among Malaysians.

## Materials and methods

### Apparatus and reagent

Glassware and plastic containers were soaked in 2 % nitric acid and left overnight before they were rinsed thoroughly with ultrapure water. All reagents used were of analytical grade. Ultrapure water was obtained from Elga, Ultra Pure Water System, Maxima.

### Sampling and sample preparation

This was a cross-sectional descriptive study, and sample size for sampling of seafood is based on statistical calculation using prevalence of 48 % of marine fish contaminated with mercury higher than the Malaysian guideline value (Agusa et al. 2005a). The formulation used was *N* = (((*Z*_*α*_^2^P (1-P)) / *E* ^2^), where *Z* = 1.96 (based on 95 % cumulative interval (CI), *E* = maximum tolerance error (5 %), and *α* = 0.05 at 95 % CI. A minimum number of 383 seafood samples were required for analysis purposes. The selection of seafood were based the most popular/consumed seafood by local population identified from results of food dietary survey conducted among 3,500 subjects in Peninsular Malaysia (Nurul Izzah et al. unpublished results) (Table 1).

**Table 1:** Most preferred seafood by population of Peninsular Malaysia

No.	Types of seafood^a^	Preference (%)
1	Indian mackerel	70.9
2	Prawn/shrimp	26.6
3	Yellowtail scad	26.2
4	Black pomfret	22.6
5	Tuna/kawakawa/bonito	21.8
6	Squid/octopus	21.3
7	Hairtail scad	20.9
8	Spanish mackerel	20.9
9	Red snapper	14.7
10	Threadfin bream	11.2
11	Stingray	10.6
12	Freshwater catfish	7.3
13	Barramundi	7.2
14	Croaker	5.4

Identified from results of food dietary survey (3-day record) conducted among 3,500 subjects in Peninsular Malaysia (Nurul Izzah et al., unpublished results)

^a^Based on common name given by study subjects

**Fig. 1:**
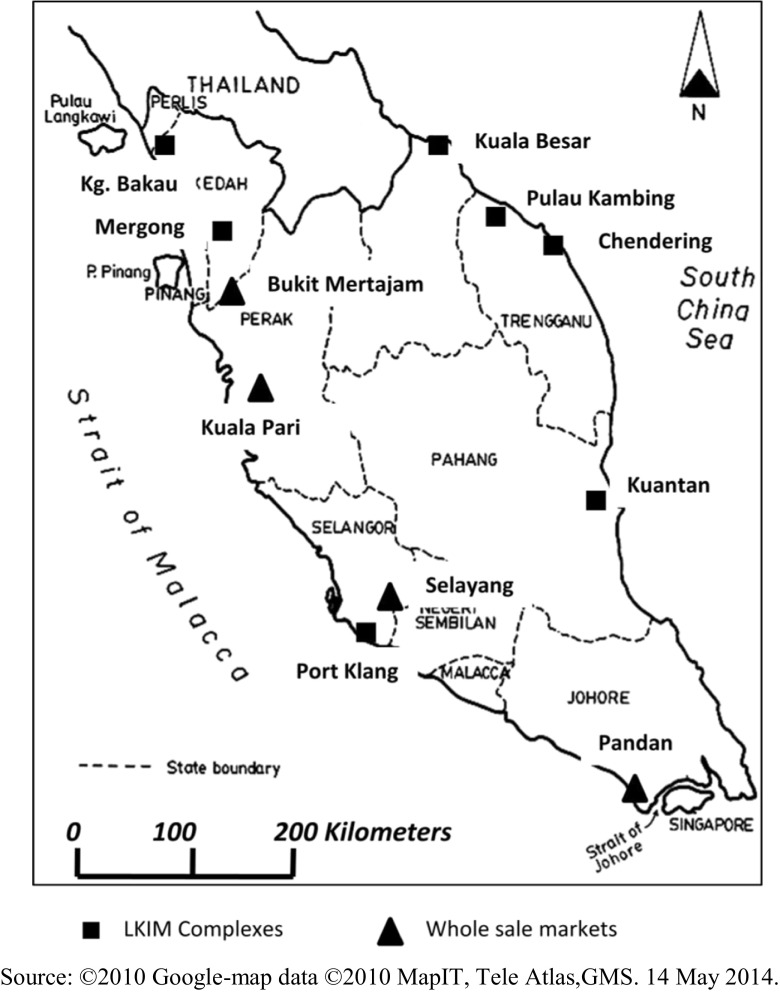
Location map of sampling stations in states of Peninsular Malaysia

Samples were purchased from six selected major fish landing complexes of Fisheries Development Authority of Malaysia (LKIM) and from five wholesale wet markets throughout Peninsular Malaysia. Two fish landing complexes were in the west coast (at Port Klang and Mergong) while the other four complexes were located along the east coast of Peninsular Malaysia (at Kuala Besar, Pulau Kambing, Chendering, and Kuantan). The five major wholesale wet markets were located at Kampong Bakau, Bukit Mertajam, Kuala Pari, and Selayang, while Pandan was the only wet market located south of Peninsular Malaysia (Fig. 1). Seafood samples were collected from the first three fishing boats that landed at the LKIM complexes on the sampling day. At the wholesale wet markets, samples were collected from three randomly selected business units. Sampling was conducted from June to December 2009. A total of 394 seafood samples were collected during three successive visits to each location, and this paper reported results of mercury determination in marine fish samples only which consist of 297 number of samples.

Total length and weight of the fish samples were measured to the nearest millimeter and gram (Table 2). Fish samples were packed in polyethylene bags, labeled, and put into an icebox before they were transported to the laboratory. In the laboratory, the samples were kept frozen at −21 °C. For sample preparation, the fish samples were thawed at room temperature. The edible portion of fish was filleted, cut into small pieces, and homogenized. The homogenized muscles were then dried in the laboratory oven at 65 °C to constant dry weight and ground using mortar.

**Table 2:** List of most preferred marine fish collected form Fisheries Development Authority of Malaysia (LKIM) fish landing complexes and whole-sale market in Peninsular Malaysia

No.	Groups/family/species	Common name	No of samples	Size range (cm)	Weight range (kg)	*Main food/feeding habits/TL
	Pelagic fish					
	(a) *Carangidae* (80)					
1	*Selaroides leptolepis*	Yellowstripe scad	13	12.6–19.6	0.028–0.290	Nekton, zooplankton, zoobenthos/predator/3.5
2	*Selar boops*	Oxeye scad	3	19.0–24.8	0.0090–0.212	Zooplankton, zoobenthos/variables/3.5
3	*Selar crumenopthalmus*	Bigeye scad	1	23.8	0.186	Nekton, zooplankton, zoobenthos, detritus/predators/4.1
4	*Atule mate*	Yellowtail scad	4	23.1–26.3	0.150–0.224	Nekton, zooplankton, zoobenthos, plants/predators/4.5
5	*Caranx sexfasciatus*	Bigeye trevally	1	22.7	0.140	Nekton/predators/4.5
6	*Seriola dumerili*	Greater amberjack	1	19.6	0.106	Nekton, zooplankton, zoobenthos/predators/4.5
7	*Decapterus kurroides*	Redtail scad	4	18.5–26.8	0.066–0.224	Zooplankton/3.4
8	*Decapterus muruadsi*	Round scad	7	18.1–36.2	0.071–0.294	Zooplankton/3.4
9	*Decapterus russelli*	Slender scad	10	16.5–30.1	0.052–0.420	Nekton, zooplankton, plants zoobenthos/variables/3.7
10	*Decapterus macrosoma*	Shortfin scad	1	–	–	Zooplankton/3.4
11	*Megalaspis cordyla*	Torpedo scad	20	22.2–34.3	0.101–0.300	Nekton, zooplankton, zoobenthos, plants detritus/predators/4.4
12	*Parastromateus niger*	Black pomfret	15	15.6–40.3	0.082–1.466	Plants, zooplankton, zoobenthos/2.9
	(b) *Scrombidae* (97)					
13	*Rastrelliger kanagurta*	Indian mackerel	13	13.4–24.3	0.200–0.360	Nekton, zooplankton, zoobenthos, plants detritus/predators/3.2
14	*Rastrelliger faughni*	Faughn’s mackerel	6	19.0–21.8	0.100–0.180	Zooplankton/3.4
15	*Rastrelliger brachysoma*	Indo-Pacific mackerel	3	16.6–25.5	0.050–0.280	Zooplankton, plants/2.7
16	*Scomber australasicus*	Blue mackerel	18	17.0–22.4	0.040–0.152	Nekton, zooplankton, zoobenthos/predators/4.2
17	*Scomberomorus guttatus*	Indo-Pacific king mackerel	12	29.6–55.5	0.176–1.066	Nekton, zoobenthos/predators/4.3
18	*Scomberomorus commerson*	Narrowbarred Spanish mackerel	14	40.1–85.5	0.394–4.550	Nekton, zooplankton, zoobenthos/predator/4.5
19	*Gymnosarda unicolor*	Dogtooth tuna	10	19.8–49.5	0.082–5.000	Nekton/predator/4.5
20	*Sarda orientalis*	Striped bonito	6	25.5–48.0	0.232–1.600	Nekton, zoobenthos/predator/4.2
21	*Thunnus tonggol*	Longtail tuna	8	17.2–50.0	0.082–1.733	Nekton, zoobenthos/predator/4.5
22	*Auxis thazard thazard*	Frigate tuna	2	29.6–31.2	0.334–0.412	Nekton, zoobenthos/predator/4.3
23	*Euthymus affinis*	Kawakawa	5	29.4–69.3	0.370–4.733	Nekton, zoobenthos/predator/4.5
	Demersal fish					
	(c) *Lutjanidae* (24)					
24	*Lutjanus argentimaculatus*	Mangrove red snapper	3	38.3–38.5	1.033–1.175	Nekton, zooplankton, zoobenthos/predator/3.6
25	*Lutjanus gibbus*	Humpback red snapper	1	20.2	0.148	Nekton, zoobenthos/predator/3.6
26	*Lutjanus sebae*	Emperor red snapper	11	18.1–62.0	0.102–3.300	Nekton, zooplankton, zoobenthos/predator/4.3
27	*Lutjanus malabaricus*	Malabar blood snapper	5	27.9–49.75	0.394–1.175	Nekton, zoobenthos/predator/4.5
28	*Lutjanus russellii*	John’s snapper	4	66.0	4.050	Detritus, nekton, zoobenthos/predator/4.3
	(d) *Latidae* (15)					
29	*Lates calcarifer*	Giant sea perch	11	26.6–71.6	0.046–4.650	Nekton, zooplankton, zoobenthos/predator/4.4
30	*Psammoperca waigiensis*	Waigeu sea perch	4	34.6–38.3	0.396–0.875	Nekton, zoobenthos/predator/4.0
	(e) *Dasyatidae* (25)					
31	*Himantura gerrardi*	Sharpnose stingray	10	21.4–116.3	0.208–2.880	Zoobenthos/predator/3.7
32	*Neotrygon kuhlii*	Bluespotted stingray	7	45.7–114.0	0.510–4.300	Nekton, zoobenthos/3.2
33	*Dasyatis zugei*	Pale-edged stingray	5	40.7–147.3	0.188–1.157	Zoobenthos/predator/3.5
34	*Himantura uarnak*	Honeycomb stingray	3	131.6–142.0	2.266–3.040	Nekton, zoobenthos/predator/3.6
	(f) *Sciaenidae* (25)					
35	*Chrysochir aureus*	Reeve’s croaker	3	19.0–25.4	0.074–0.220	Zoobenthos/predator/3.5
36	*Otolithoides ruber*	Tigertooth croaker	6	13.0–24.2	0.04–0.113	Zoobenthos/predator/3.6
37	*Nibea soldado*	Soldier croaker	15	15.3–21.6	0.041–0.274	Zoobenthos, nekton/predator/4.0
38	*Otolithoides biauritus*	Bronze croaker	1	20.9	0.132	Zoobenthos, nekton/predator/4.1
	(g) *Nemipteridae* (31)					
39	*Nemipterus bathybius*	Yellowbelly threadfin bream	6	15.7–33.7	0.063–0.516	Zoobenthos, nekton/predator/4.0
40	*Nemipterus japonicus*	Japanese threadfin bream	11	16.9–29.2	0.063–0.212	Zoobenthos, nekton/predator/3.8
41	*Nemipterus furcosus*	Forktail threadfin bream	3	18.2–21.4	0.102–0.162	Zoobenthos, nekton/predator/3.6
42	*Nemipterus thosaporni*	Threadfin bream	4	17.0–24.0	0.059–0.210	Not available/3.7
43	*Nemipterus tambuloides*	Fivelined threadfin bream	2	17.5–21.4	0.086–0.109	Zoobenthos, nekton/predator/4.0
44	*Nemipterus nematophorus*	Doublewhip threadfin bream	2	16.1–25.6	0.058–0.154	Not available/3.7
45	*Nemipterus marginatus*	Red filament threadfin bream	2	23.0–25.5	0.110–0.240	Zoobenthos/predator/3.5
46	*Nemipterus nemurus*	Redspine threadfin bream	1	17.8	0.096	Zoobenthos, nekton/predator/4.0

Source: http://www.fishbase.us. Nekton—the division of the pelagic population that comprises of the free-swimming animals. They are capable of withstanding the force of the ocean current and are able to travel over long distances. Fish, squids, cetaceans, pinnipeds, sea snakes, turtles, and penguins constitute the nekton group. Zooplankton—animal constituent of plankton; mainly small crustaceans and fish larvae. Free-swimming aquatic animals, essentially independent of water movements. Zoobenthos—the invertebrate animals that live in or on the seabed, including the intertidal zone. Plants—phytoplankton and other plants. Detritus—non-living particulate organic material (as opposed to dissolved organic material). It typically includes the bodies or fragments of dead organisms as well as fecal material

Tropic levels (TLs):

2.0–2.1 (mean 2.02)—pure herbivores

2.1 < TL < 2.9 (mean 2.5)—omnivores with a preference for vegetable material

2.9 < TL < 3.7 (mean 3.4)—omnivores with a preference for animal material (feeding on a variety of prey)

3.7 < TL < 4.0 (mean 3.85)—carnivores with a preference for decapods and fish

4.0 < TL < 4.5 (mean 4.32)—carnivores with a preference for fish cephalopods

(Stergiou and Karpouzi 2002)

The food items and feeding habits of both the pelagic and demersal fishes were referenced from the Global Information System on Fishes at the website: http://www.fishbase.us (Table 2). Most of the fish sampled in this study were classified as predatory, which live by killing and eating upon other fish or animals. Only five species (redtail scad, round scad, shortfin scad, Faughn’s mackerel, and Indo-Pacific mackerel) that were captured in this study were non-predators. They fed on zooplankton, phytoplankton, and other plants. Information on tropic levels (TL) that expressed the position of a species in a marine food web was gathered from the same website. In the marine ecosystem, TL of consumers generally ranges between 2.0 for species feeding exclusively on plants or detritus and 5.5 for carnivores (Stergiou and Karpouzi 2002).

### Digestion procedures

Samples for mercury analysis (including blanks) were digested in a microwave digestion system (Multiwave 3000—Anton Paar). Dried fish samples were weighed accurately into the digestion vessels for 0.5 g. A total of 5.0-ml concentrated nitric acid and 2.0 ml of hydrogen peroxide were added to each vessel. The vessels were sealed and placed into the rotor for microwave digestion. The power profile used for the digestion of samples with the Multiwave was as follows: During the first phase, the power of the digestion system was set at 600 W, followed by 5-min ramping and holding, respectively. At the second phase, the power was increased to 1,400 W followed by 5-min ramping and 10-min holding time. Finally, at phase 3, the power was turned to zero with holding time of 15 min. After the digestion process, samples with clear solutions were filtered through a 0.45-μm acid-resistant membrane. The solution was transferred to a 25-ml volumetric flask and diluted with ultrapure water. The analytical reagent blanks were also prepared in the same manner but without the dried fish samples.

### Mercury analysis

Mercury was analyzed by the cold vapor atomic absorption spectrometry (AAS) technique using the PerkinElmer Flow Injection Mercury System (FIMS) instrument equipped with FIMS-400 and a programmable sample dispenser following the method of Mohd Fairulnizal et al. (1998). Stock standard solution of mercury, 1,000 μg/ml, was obtained from PerkinElmer. A sub-stock solution of 10 mg/l was prepared by dilution of the stock standard solution. The working standard solutions of 0, 2, 5, and 10 μg/l were prepared by further dilution of the sub-stock solution. These working standards were prepared fresh daily. A linear range calibration method was used and the correlation coefficient was controlled at *r* = 0.9995. Detection limit was based on the mercury concentration corresponding to three times the standard deviation of ten reagent blanks, which was 0.72 μg/l. The analysis was validated by injecting of two different concentrations of mercury standard solutions utilized as quality control between each ten injection of samples, and the acceptable range was set between 85 and 110 %. Analytical control was accompanied by analysis of reagent blanks and standard reference samples (NIST SRM® 1946—Lake Superior Fish Tissue). Average recovery of reference standards reached 90.7 %. The results were expressed in dry weight basis.

In order to compare the results with the national and international guidelines for the purpose of public health perspective, it was necessary to convert mercury concentrations in fish samples to a wet basis values using the formula: Dry weight concentration = wet weight concentration × (100/100 moisture percentage). The calculation for the amount of moisture content was calculated based on the works of Tee et al. (1997) and Nurnadia et al. (2011). The results were then grouped into five categories, following Chvojka et al. (1990) as cited by Al-Majed and Preston (2000). They described mercury in wet weight of fish from 0.05 to 0.15 μg/g as very low, 0.15–0.25 μg/g as low, 0.25–0.35 μg/g as medium, 0.35–0.45 μg/g as high, and above 0.45 μg/g as very high. The recommended guideline levels by the joint FAO/WHO Expert Committee on Food Additives (FAO/WHO 2006) was set at 0.5 mg/kg methylmercury in fish. In Malaysia, under the Fourteenth Schedule of Regulation 38, Malaysian Food Regulation 1985 (Food Act 1983, (Act 281) and Regulations 2006), the maximum permitted proportion of methylmercury was set at the same level.

### Statistical analysis

Data was cleaned and checked for discrepancies before analysis. The initial descriptive statistical analysis showed that the data was not normally distributed due to the existence of the outliers. Hence, non-parametric statistics were used. The medians, interquartile range, and percentile range were calculated using SPSS (version 11.5 for Windows, 2002, SPSS Inc.). The statistical significance of difference was assessed using Mann-Whitney’s (MW) test for two groups and Kruskall-Wallis’s (KW) test for three groups or more. The correlation coefficients were studied using Spearman correlation analysis. The level for significance was designated as *p* < 0.05.

## Results

A total of 297 samples that composed of 46 species of marine fish collected from selected major LKIM fish landing ports and wholesale markets in Peninsular Malaysia are shown in Table 2. A number of 177 samples of pelagic fish were collected in this study. This group of fish live near the surface or in the water column of coastal and ocean. They were then classified into two different families: 80 of *Carangidae* and 97 of *Scrombidae*. The remaining 120 fish samples were demersal fish that live on or near the bottom of the sea or ocean. There were five families of demersal fish collected in this study, which were 24 of *Lutjanidae*, 15 of *Latidae*, 25 of *Dasyatidae*, 25 of *Sciaenidae*, and 31 of *Nemipteridae*.

The size of fish in the samples varied. The *Carangidae*, *Sciaenidae*, and *Nemipteridae* were small-sized fish with body length ranging from 12 to 30 cm and with weight of less than 0.5 kg. Other family groups covered a relatively wide size range that composed of small, medium to large-sized fish; the smallest weighed 40 g and the largest 5 kg. Generally, the larger fish were Spanish mackerels, tuna, red snapper, sea perch, and stingray.

The TL for fish captured in this study ranged from 2.7 to 4.5. More than half (66.7 %) of the samples had TL range from 3.7 to 4.5, which indicated that most of the samples captured were carnivores or large pelagic. Another 32.3 % had TL between 2.9 and 3.7 with a mean value of 3.4. This group was omnivorous that fed on a variety of prey. Only 1 % of the samples fed on vegetable materials.

Total mercury levels in marine fish sampled from the fish landing ports and the wholesale markets in Peninsular Malaysia are summarized in Table 3. Mercury levels of 46 marine fish species ranged from 0.055 to 2.537 mg/kg of dry weight. Significant variations of mercury levels exist in different species (*χ*_KW_^2^ = 103.581; *p* < 0.001). Among pelagic fish, the median for mercury levels was higher (>0.5 mg/kg) in scad (*Selar boops*) and bonito (*Sarda orientalis*). While for the demersal fish, the highest mercury levels were shown in John’s snapper (*Lutjanus ruselli*), mangrove red snapper (*Lutjanus argentimaculatus*), and doublewhip threadfin bream (*Nemipterus nematophorus*). Mercury levels were significantly higher in higher tropic level fish (*χ*_KW_^2^ = 7.787; *p* < 0.02).

**Table 3:** Total mercury levels in marine fish sampled from the LKIM complexes and wholesale market in Peninsular Malaysia

No.	Groups/family/species	Common name	Number	Total mercury (mg/kg dry weight (DW))				
				Median	IQR	Min	Max	Range
	Pelagic fish							
	*Carangidae* (80)							
1	*Selaroides leptolepis*	Yellowstripe scad	13	0.252	0.125	0.138	2.137	1.999
2	*Selar boops*	Oxeye scad	3	0.555	–	0.305	0.719	0.414
3	*Selar crumenopthalmus*	Bigeye scad	1	0.298	–	–	–	–
4	*Atule mate*	Yellowtail scad	4	0.458	0.304	0.371	0.746	0.375
5	*Caranx sexfasciatus*	Bigeye trevally	1	0.293	–	–	–	–
6	*Seriola dumerili*	Greater amberjack	1	0.203	–	–	–	–
7	*Decapterus kurroides*	Redtail scad	4	0.272	0.263	0.186	0.535	0.349
8	*Decapterus muruadsi*	Round scad	7	0.317	0.171	0.173	0.535	0.362
9	*Decapterus russelli*	Slender scad	10	0.195	0.108	0.078	0.304	0.226
10	*Decapterus macrosoma*	Shortfin scad	1	0.354	–	–	–	–
11	*Megalaspis cordyla*	Torpedo scad	20	0.319	0.198	0.202	0.913	0.711
12	*Parastromateus niger*	Black pomfret	15	0.242	0.121	0.158	0.518	0.360
		Total	80	0.291	0.153	0.078	2.137	2.059
	*Scrombidae* (97)							
13	*Rastrelliger kanagurta*	Indian mackerel	13	0.180	0.066	0.055	0.454	0.399
14	*Rastrelliger faughni*	Faughn’s mackerel	6	0.357	0.246	0.254	0.753	0.499
15	*Rastrelliger brachysoma*	Indo-Pacific mackerel	3	0.261	–	0.258	0.332	0.074
16	*Scomber australasicus*	Blue mackerel	18	0.269	0.065	0.152	0.610	0.458
17	*Scomberomorus guttatus*	Indo-Pacific king mackerel	12	0.262	0.355	0.159	0.983	0.824
18	*Scomberomorus commerson*	Narrowbarred Spanish mackerel	14	0.368	0.953	0.153	1.378	1.226
19	*Gymnosarda unicolor*	Dogtooth tuna	10	0.342	0.456	0.216	1.518	1.302
20	*Sarda orientalis*	striped bonito	6	0.543	1.048	0.200	0.179	1.592
21	*Thunnus tonggol*	Longtail tuna	8	0.358	0.173	0.178	0.565	0.387
22	*Auxis thazard thazard*	Frigate tuna	2	0.237	–	0.207	0.266	0.059
23	*Euthymus affinis*	Kawakawa	5	0.289	–	0.226	0.322	0.096
		Total	97	0.293	0.190	0.055	1.792	1.737
	Demersal fish							
	*Lutjanidae* (24)							
24	*Lutjanus argentimaculatus*	Mangrove red snapper	3	0.856	–	0.317	0.950	0.633
25	*Lutjanus gibbus*	Humpback red snapper	1	0.436	–	–	–	–
26	*Lutjanus sebae*	Emperor red snapper	11	0.334	0.516	0.173	1.810	1.637
27	*Lutjanus malabaricus*	Malabar blood snapper	5	0.413	0.366	0.290	0.723	0.433
28	*Lutjanus russellii*	John’s snapper	4	1.366	–	–	–	–
		Total	24	0.465	0.566	0.173	2.666	2.493
	*Latidae* (15)							
29	*Lates calcarifer*	Giant sea perch	11	0.537	0.436	0.255	1.408	1.153
30	*Psammoperca waigiensis*	Waigeu sea perch	4	0.532	0.165	0.398	0.601	0.203
		Total	15	0.537	0.267	0.255	1.408	1.153
	*Dasyatidae* (25)							
31	*Himantura gerrardi*	Sharpnose stingray	10	0.384	0.741	0.206	1.432	1.226
32	*Neotrygon kuhlii*	Bluespotted stingray	7	0.492	1.251	0.226	2.537	2.311
33	*Dasyatis zugei*	Pale-edged stingray	5	0.548	0.509	0.233	0.905	0.672
34	*Himantura uarnak*	Honeycomb stingray	3	0.425	–	0.204	2.517	2.313
		Total	25	0.492	0.740	0.204	2.537	2.333
	*Sciaenidae* (25)							
35	*Chrysochir aureus*	Reeve’s croaker	3	0.498	–	0.282	0.733	0.451
36	*Otolithoides ruber*	Tigertooth croaker	6	0.421	0.423	0.283	0.775	0.492
37	*Nibea soldado*	Soldier croaker	15	0.424	0.132	0.181	1.227	1.046
38	*Otolithoides biauritus*	Bronze croaker	1	0.069	–	–	–	–
		Total	25	0.424	0.217	0.069	1.227	1.158
	*Nemipteridae* (31)							
39	*Nemipterus bathybius*	Yellowbelly threadfin bream	6	0.383	0.328	0.263	0.753	0.490
40	*Nemipterus japonicus*	Japanese threadfin bream	11	0.464	0.724	0.213	1.206	0.993
41	*Nemipterus furcosus*	Forktail threadfin bream	3	0.642	–	0.371	0.918	0.547
42	*Nemipterus thosaporni*	Threadfin bream	4	0.570	0.659	0.402	1.244	0.842
43	*Nemipterus tambuloides*	Fivelined threadfin bream	2	0.426	–	0.397	0.454	0.057
44	*Nemipterus nematophorus*	Doublewhip threadfin bream	2	1.211	–	0.858	1.563	0.704
45	*Nemipterus marginatus*	Red filament threadfin bream	2	0.244	–	0.207	0.281	0.074
46	*Nemipterus nemurus*	Redspine threadfin bream	1	0.298	–	–	–	–
		Total	31	0.454	0.459	0.207	1.563	1.356

Comparison of mercury levels for different fish species: *χ*_KW_^2^ = 109.596, *p* = 0.000

*IQR* interquartile range

**Fig. 2:**
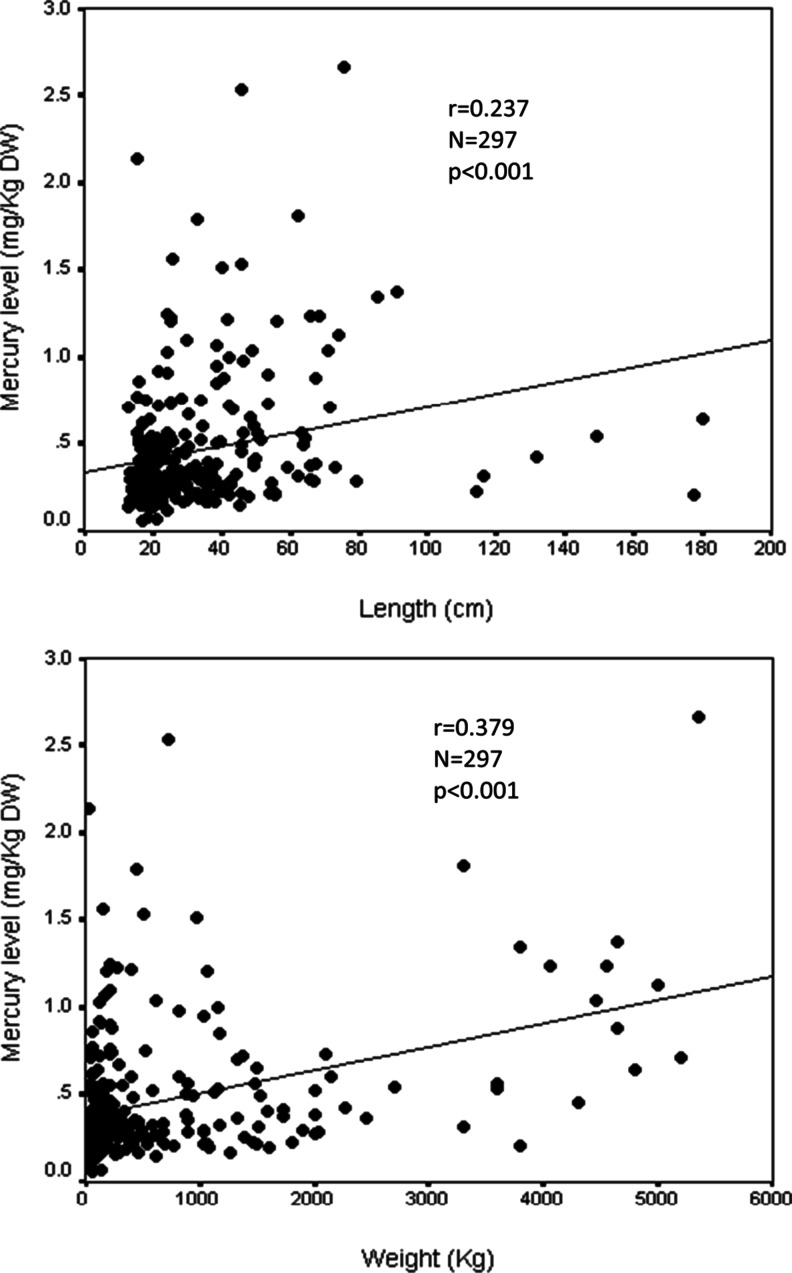
Relationship between total mercury levels (mg/kg dry weight), length, and weight of marine fish samples from Peninsular Malaysia

The median of mercury levels for demersal fish (0.460 ± 0.414 mg/kg dry weight) was significantly higher (*χ*_MW_^2^ = 5401.0; *p* < 0.001) compared to the pelagic fish (0.292 ± 0.169 mg/kg of dry weight) (Table 4). Mercury level was significantly lower (*χ*_KW_^2^ = 7.787; *p* < 0.02) in herbivorous when compared to the omnivorous and carnivorous fish. Among the family groups, mercury levels were significantly higher (*χ*_KW_^2^ = 46.122; *p* < 0.001) in *Latidae*, followed by *Dasyatidae*, *Lutjanidae*, and *Nemipteridae*. No significant differences (*χ*_MW_^2^ = 1,863.0; *p* = 0.274) were shown for mercury levels between local and imported fish, different coastal regions (west, east, and south) (*χ*_KW_^2^ = 679.0; *p* = 0.712), and sampling points (fish landing ports and wet markets) (*χ*_MW_^2^ = 10,114.0; *p* = 0.738). However, mercury levels in marine fish sampled from different sampling locations showed significant differences at *p* = 0.014 (*χ*_KW_^2^ = 22.263). Higher mercury levels were found in fish sampled from Port Klang (0.563 ± 0.509 mg/kg of dry weight), Kuala Besar (0.521 ± 0.415 mg/kg of dry weight), and Pandan (0.380 ± 0.481 mg/kg dry weight) compared to other locations. Mercury levels in bigger fish were significantly higher (*χ*_MW_^2^ = 6,642.0; *p* = 0.002) compared to the smaller ones. Scatter plots (Fig. 2) showed that mercury levels were positively correlated with weight and length of the fish, with significant Spearman correlation coefficients of 0.237 and 0.297, respectively.

**Table 4:** Comparison of mercury levels in marine fish collected from Peninsular Malaysia at different factors

No.	Factors	Number	Median (IQR)	(*χ* ^2^) *p* value
1	Habitats			
	Pelagic	170	0.292 (0.169)	^MW^5401.0 (0.000)
	Demersal	118	0.460 (0.414)	
2	Feeding habits			
	Herbivorous	19	0.258 (0.118)	^KW^7.787 (0.020)
	Omnivorous	109	0.334 (0.322)	
	Carnivorous	160	0.354 (0.250)	
3	Family group			
	*Carangidae*	79	0.291 (0.153)	^KW^46.122 (0.000)
	*Scrombidae*	91	0.293 (0.190)	
	*Lutjanidae*	22	0.465 (0.566)	
	*Latidae*	15	0.537 (0.267)	
	*Dasyatidae*	25	0.492 (0.740)	
	*Sciaenidae*	25	0.424 (0.217)	
	*Nemipteridae*	31	0.454 (0.459)	
4	Origins			
	Local	231	0.332 (0.275)	^MW^1863.0 (0.274)
	Import	19	0.359 (0.237)	
5	Coastal			
	West coast	97	0.330 (0.255)	^KW^0.679 (0.712)
	East coast	158	0.333 (0.272)	
	South	33	0.380 (0.481)	
6	Sampling points			
	LKIM fish landing complexes	150	0.354 (0.298)	^MW^10114.0 (0.738)
	Wholesale wet market	138	0.334 (0.263)	
7	Sampling locations			
	Selayang	25	0.295 (0.253)	^KW^22.263 (0.014)
	Klang	20	0.563 (0.509)	
	Kuala Pari	37	0.356 (0.292)	
	Bukit Mertajam	14	0.348 (0.156)	
	Kuala Perlis	29	0.306 (0.185)	
	Mergong	33	0.320 (0.188)	
	Kuala Besar	20	0.521 (0.415)	
	Pandan	33	0.380 (0.481)	
	Kuantan	36	0.301 (0.142)	
	Chendering	15	0.285 (0.142)	
	Pulau Kambing	26	0.321 (0.222)	
8	Body length of fish			
	Body length <20 cm	92	0.311 (0.223)	^MW^6642.0 (0.002)
	Body length ≥20 cm	187	0.354 (0.306)	

Kruskal-Wallis (KW) and Mann-Whitney (MW) *U* test were applied

*IQR* interquartile range

**Fig. 3:**
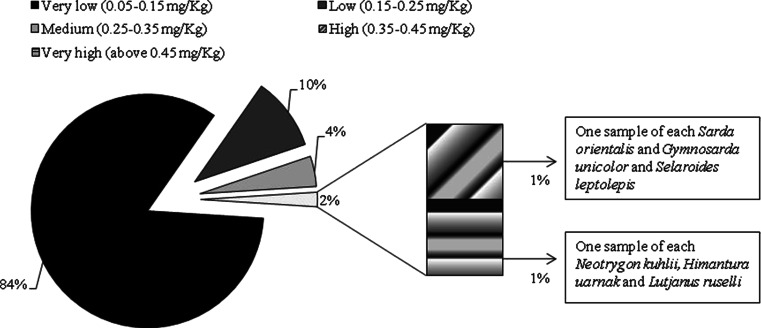
The distribution of total mercury in fish based on categories by Chvojka et al. (1990) in their study: mercury in snapper from the New South Wales Coast, Australia. These categories were cited by Al-Majed and Preston (2000) in their study of mercury content in zooplankton and fish tissue collected from Kuwait Territorial Waters

The distribution of total mercury in fish based on categories by Chvojka et al. (1990) is shown in Fig. 3. The results from this study indicated that most of the samples (83.7 %) had very low levels of mercury followed by 10.1 % with low mercury levels. Another 4.2 % of the samples had medium levels of mercury, and 1 % had high levels. Only 1 % or three samples of bluespotted stingray (*Neotrygon kuhlii*), honeycomb stingray (*Himantura uarnak*), and John’s snapper (*Lutjanus ruselli*) had very high mercury levels. The latter two samples exceeded the guidelines of 0.5 mg/kg. However, considering that 95 % or more of total mercury in the edible portions of fish and other seafood is in the form of methylmercury (Li et al. 2009; Khaniki et al. 2005), only one sample (median mercury level in *Lutjanus ruselli*, 0.5012 mg/kg) exceeded the guidelines. None of the samples exceeded the guidelines if the ratio of methylmercury to total mercury ranged from 70 to 83 % were considered (Hajeb et al. 2010).

Based on the five categories, this study has also identified few samples of fish with high mercury levels, two samples of tuna (*S. orientalis* and *Gymnosarda unicolor*) and one sample of yellowstripe scad (*Selaroides leptolepis*). For medium levels, one sample of snapper (*Lutjanus sebae*), four samples of narrow-barred Spanish mackerel (*Scomberomorus commerson*), one sample of barramundi (*Lates calcarifer*), two samples of stingray (*Himantura gerrardi and Neotrygon kuhlii*), two samples of tuna (*G. unicolor and S. orientalis*), and one sample of each thread-fin bream (*N. nematophorus*) and soldier croaker (*S. orientalis*).

## Discussion

The present study provides data on total mercury levels in marine fish tissue sampled from both the fish landing ports and the wholesale wet markets throughout Peninsular Malaysia. This study has several advantages whereby mercury was determined in a wide range of fish species (46 marine fish species) captured from the different geographical areas over different periods, with three visits for each sampling location within a 6-month time (from June to December 2009). Several interesting findings can be reached from this study: The data indicated that mercury levels differed significantly among the different families and species of fish. It also showed that mercury were found to be significantly higher in omnivorous and carnivorous food feeding fish compared to the herbivorous. The mercury levels were also significantly higher in demersal fish when compared to the pelagic fish. Fish captured from highly anthropogenic activity areas showed higher mercury level, and positive correlation were shown between mercury concentrations with fish size. Each of these factors was discussed below.

The results from this study concurred with other Malaysian studies (Table 5), in that most of the mercury levels in different species of fish were lower than the national and international permissible limits. Hajeb et al. (2009) reported comparable results of mercury levels in Indo-Pacific mackerel (0.45 ± 0.056 μg/g dry weight (DW)), black pomfret (0.13 ± 0.15 μg/g DW), and longtail tuna (0.5 ± 0.71 μg/g DW) as compared to this study. However, they reported lower mercury levels for narrow-barred Spanish mackerel (0.04 ± 0.02 μg/g DW), giant perch (0.10 ± 0.06 μg/g DW), and sardines (0.00 ± 0.02 μg/g DW). Later, they reported extremely higher mercury levels in *Rastrelliger brachysoma* (0.229–0.778 μg/g WW) and *Thunnus tonggol* (0.225–0.914 μg/g WW) compared to this study (Hajeb et al. 2010). Results from Kamaruzaman et al. (2011) showed that the mercury levels were ten times lower in *Nemipterus japonicas* (0.012 ± 0.008 μg/g DW), *L. sebae* (0.015 ± 0.001 μg/g DW), and *Otolithes ruber* (0.017 ± 0.003 μg/g DW) compared to this current study. Alina et al. (2012) reported much lower results of mercury levels in Japanese threadfin bream (0.004–0.0065 μg/g wet weight (WW)), Malabar red snapper (0.0021–0.0054 μg/g WW), Spanish mackerel (0.002–0.0022 μg/g WW), and Indian mackerel (0.001–0.0011 μg/g WW). They also found that the mercury levels in pelagic fish were lower than the levels in demersal fish. Mercury level in *Nemipterus furcosus* is relatively higher in this study (0.642 μg/g DW), and the results are similar as reported by Agusa et al. (2007) (0.67 μg/g DW). They also reported similar results for Torpedo scad (0.21–0.27 μg/g DW) and bigeye scad (0.11–0.36 μg/g DW). None of these previous studies reported on the mercury levels in *N. nematophorus*, whereas in this study, the level was among the highest for family *Nemipteridae*. Nevertheless, only two samples were captured in this study (Table 3).

**Table 5:** Recent publications of mercury analysis in marine fish in Malaysia

Sampling locations	Species and no. of samples	Significant findings	References
Retail outlet in Selangor and fishes originated from the South China Sea	A total of 69 marine fish samples from 12 different species	*Thunnus tonggol* (0.5 μg/g DW) and *Rastrelliger brachsoma* (0.45 μg/g DW) had the highest level of mercury compared to other species.	Hajeb et al. (2009)
Fishermen boat at fish landings in Kuantan, Chendering, and Kuala Perlis	A total of 69 samples of short-bodied mackerel and longtail tuna	Total mercury in all samples ranged from 0.180 to 1.460 μg/g DW. Samples of both species from east coast showed higher levels compared to the west coast.	Hajeb et al. (2010)
Local LKIM fish market from Johor, Melaka, and Negeri Sembilan	A total of 162 demersal marine fish from five species	Range of Hg in muscle tissue were between 0.012 and 0.019 μg/g DW	Kamaruzaman et al. (2011)
Ten identified fish landing areas along the Strait of Malacca	A total of 60 marine fish from 12 species of most popular and preferred by local consumers	Hg levels in demersal fish ranging from 0.0017 to 0.0065 ppm WW. In pelagic fish the range was between 0.001 and 0.0065 ppm. None of the samples exceeded the permitted levels.	Alina et al. (2012)
Cabang Tiga Kelantan, Kuala Terengganu, Mersing, Parit Jawa, Port Dickson, and Langkawi	A total of 102 samples from 13 species of marine fish	Mercury levels in fish muscle ranged from less than 0.05 to 0.67 μg/g DW. The highest Hg was determined in Fork-tailed threadfin bream and bigeye scads.	Agusa et al. (2007)
Pulau Tuba, Langkawi	A total of 76 marine fish samples from eight different species	Mercury levels in fish were very much lower compared to the permissible limits set by the FAO/WHO in 1984, ranging from 0.02 to 0.08 ppm DW	Irwandi and Farida (2009)

Total mercury median concentrations varied significantly (*p* < 0.05) among different fish species tested. Mercury accumulates into fish tissue through the food chain whereby it transfers between aquatic plants and aquatic animals, from sediment, as well as from the water environments (Bidone et al. 1997). Methylmercury is a major fraction of total mercury concentrations accumulated into fish tissue where it ranges from 85 to 97 % (Bidone et al. 1997). Highest percentage of mercury as methylmercury in ray species was found in muscle tissue and accounting to nearly 100 % of mercury present as methylmercury (Horvat et al. 2014). The indirect bioaccumulation process is a phenomena in which a mercury substance accumulates into fish tissue based on its tropic level in a food chain (Bidone et al. 1997). The variability of fish food is based on their habitat, which may be demersal, bentho-pelagic, pelagic, bathy-demersal, and reef-associated with fish living and feeding on or near coral reefs (Stergiou and Karpouzi 2005). Tropic level expresses the position of a species in the marine food web, and its estimation requires knowledge of what each species feeds on and in what quantities (Stergiou and Karpouzi 2005). Briefly, mercury enters the food chain via phytoplankton and then accumulated as methylmercury by other links in the chain, via tropic transfer (Seixas et al. 2013).

Carnivorous species are placed at higher tropic level than non-carnivorous species in a food chain (Bidone et al. 1997; Stergiou and Karpouzi 2005). These groups of fish live and feed in the open sea and are associated with the surface or middle depths of a body of water; they are free-swimming in the seas, oceans, or open waters, and they were not associated with the bottom (Stergiou and Karpouzi 2005). It is well known that mercury concentrations in carnivorous fish with higher tropic level are higher than in herbivores, omnivores, or planktivores (Burger et al. 2001; Burger and Gochfeld 2011; Liu et al. 2014). Yet, mercury concentration in top-level predators had the highest levels compared to the bottom-dwelling fish (Burger and Gochfeld 2011). Findings from this study explained this facts where mercury levels in carnivorous fish is more than 1.3 times higher as compared to the levels in herbivorous fish. Another study conducted at Tapajos River basin in the Munduruku Reserve, Jacareacanga, Brazil, showed that the mean mercury concentrations in carnivorous fish (0.297 μg/g) was three times higher when compared to the non-carnivorous species (0.095 μg/g) (Brabo et al. 2000). An earlier study conducted at the same river basin showed the differences of up to seven times between non-carnivorous and carnivorous fish (Bidone et al. 1997). The differences were even more in fish captured from the municipality of Itaituba, Tapajos River Basin, Para, Brazil, where mercury concentrations in carnivorous fish ranged from 112.4 to 2,250 μg/g compared to the detrivorous, herbivorous, and omnivorous that ranged from 3.2 to 309.8 μg/g (dos Santos et al. 2000). Another study by Seixas et al. (2013) showed that the mud-eater iliophagous fish were the food items to the voracious predator, and they were also indirectly transmitted the methylmercury to this top predator. Ferriss et al. (2014) had developed models that capable of exploring variations in the concentrations of mercury in top pelagic predators relative to food web structure and mercury input at the base of the system. The data from this study is consistent with those previous studies where the mercury levels in fish with higher tropic level were significantly higher than in fish with lower tropic level. Mercury accumulation through the food chain resulted in higher mercury concentrations in predator fish when compared to fish with lower tropic level.

Although tropic level correlations with mercury in fish have been reported in many studies (Kinghorn et al. 2007; Evans et al. 2005; Burger et al. 2001; Brabo et al. 2000; dos Santos et al. 2000; Burger and Gochfeld 2011), it was not the only factor that affects mercury contamination in fish (Burger and Gochfeld 2011). Another important factor for mercury contamination in fish was found to be the bioaccumulation process based on its bioavailability, uptake, and toxicokinetics (Burger et al. 2001). Other additional recorded factors were physiological differences between different fish species, migration from unpolluted areas to relatively more polluted areas (Al-Majed and Preston 2000), total organic carbon, biologic activity, pH, conductance, oxygen concentration, water temperature, water level, wetland runoff (Kinghorn et al. 2007), seasons, and habitat (Saei-Dehkordi et al. 2010).

The results for total mercury in demersal fish from this study showed higher levels of mercury, of nearly two times more than in the pelagic fish. Among demersal fish, the highest values corresponded to the smaller species (*N. furcosus*, *Nemipterus thosaporni*, *N. nematophorus*), large demersal species (*L. argentimaculatus*, *Lutjanus russelli*), and benthic species (*Dasyatis zugei*, *H. uarnak*). The benthic species exhibited high concentrations of mercury than those of pelagic species. This reflected the area variations that may be due to the highest values of mercury in the marine environment where high methylation rates occurred (Arcos et al. 2002; Joiris et al. 1999). Benthic species are more exposed to higher concentrations of methylmercury in the sediment (Al-Majed and Preston 2000) and of their specific prey (Eagle-Smith et al. 2008). Methylation processes are important in the sub-thermocline waters of the open ocean where low oxygen conditions favor those organisms that transform inorganic mercury into organic forms (Arcos et al. 2002).

In addition to natural inputs, local pollution in the areas where the fish were captured could explain the concentrations of mercury for this study. Most of the fish samples in this study were of local origin, and they were mainly harvested from the South China Sea and the Strait of Malacca. The countries surrounding the South China Sea are among the most densely populated, fastest growing and until 1997, the most vibrant economies on earth. There would have been introduced wastes from large cities (sewage, industrial waste, and hydrocarbons) and agricultural runoff (nutrients, pesticides and sediment) into the sea (Morton and Blackmore 2001). Besides, the Strait of Malacca contained waste discharged from both land-based and sea-based sources (Chua et al. 2000). For example, this is the area where vessels operate, also hustles of activities from gas platforms activities as well as a consequence of accidents. High concentrations of heavy metal were reported in the waters off the southern coast of Singapore and near petroleum refineries, and it was also detected in bottom sediment, especially in areas experiencing heavy shipping traffic (Chua et al. 2000).

In this study, with mercury contamination being higher in fish samples collected from Port Klang, Selangor, it would reflect urban contamination from the large cities of Kuala Lumpur and Shah Alam, Selangor, and also from shipping activities around Port Klang itself. Mercury contamination in fish collected from Pandan Johor would have been a result of urban activities of Johor Bahru, as well as from the nearby Keppel Harbour and the main port of Singapore (Chua et al. 2000). For Kuala Besar, Kelantan, which is situated at the east coast of Peninsular Malaysia, higher mercury contamination was a result of pollution from anthropogenic activities in the South China Sea coastal waters (Liu et al. 2014), oil well activities in the surrounding area, petroleum refineries, and oil tanker movements (Morton and Blackmore 2001). Re-suspension and deposition of dissolved and particulate matter by rain were also major sources of metal pollution in Asian marginal seas (Macdonald et al. 2002; Liu et al. 2014). Understanding the migration patterns and origins of fish are also important factors as the seasonal variation in mercury probably reflects movements of fish of various sources and sizes (Gochfeld et al. 2012). It would seem that a combination of the several factors listed here were responsible for the differences of mercury body burdens between the different species of fish in this study.

A positive relationship between total mercury levels and fish size were often observed indicating that mercury levels tend to increase over time during the growth of the fish (Burger et al. 2001, 2007; Cai et al. 2006; Adams and McMichael 2007; Burger 2009; Burger and Gochfeld 2011; Gochfeld et al. 2012; Seixas et al. 2013; Horvat et al. 2014). In this study, a positive relationship between mercury content and weight was more apparent than when compared to the fish length. These relationships are influenced by the relatively slow rate of mercury eliminated when compared to the rate of its accumulation in fish tissues (Adams and McMichael 2007). Mercury elimination rates for fish tend to decrease with increasing fish body size (Adams and McMichael 2007) as elimination rate is negatively correlated with size (Trudel and Rasmussen 1997). Larger fish usually are older and have had longer time to accumulate mercury (Gochfeld et al. 2012) from their environment. Moreover, they may eat larger prey and varieties of other species of prey that are already highly contaminated with mercury (Adams and McMichael 2007; Gochfeld et al. 2012). Length (Weis 2004; Kinghorn et al. 2007; Li et al. 2009) and weight (Al-Majed and Preston 2000) were used as standard indicators of fish age and were usually used as proxy measurements for age as it was easy to determine in the field (Kinghorn et al. 2007). As a result, in older or longer or bigger fish, mercury concentration was expected to increase unless elimination took place and/or the fish migrated from the polluted area to a relatively less or unpolluted area.

The results of this study will contribute toward the baseline data and information on mercury concentration in marine fish that are commonly consumed in Peninsular Malaysia. This study has identified lower mercury levels in marine fish according to the guidelines; thus, commercial marine fish from Peninsular Malaysia is considered safe for human consumption. It would be appropriate to note that the risk from mercury contaminations could still have an impact on human health if fish were to be consumed in excessive amounts. This is especially so when one refers to the fish species found to have medium to very high levels of mercury contamination; hence, these data would be invaluable as it would provide useful information for assessment of potential health risks from mercury contamination in the populations of Peninsular Malaysia.

## Conclusion

This study evaluated mercury concentrations in 46 species of commonly consumed marine fish sampled from fish landing ports and wholesale markets in Peninsular Malaysia. The data provided information and served as baseline reference for future studies concerning mercury contaminations in marine fish for the country. The edible portion of marine fish contained mercury at low levels and were within the permissible limits by both national and international guidelines. The results from this study also showed that the total mercury concentration in marine fish was positively correlated with length and weight, in that it reflected an accumulation of mercury with time. In addition, the mercury concentrations were found to be higher in carnivorous fish when compared to the herbivorous. Mercury concentrations were also higher in demersal fish, especially the benthic species than their levels in the pelagic species. Mercury concentration in fish collected from regions with highly anthropogenic input such as large cities industrial and shipping activities were significantly higher when compared to fish collected from less contaminated regions. Thus, it explained that there was a geographical variability without underestimating the natural sources. There is a need for future studies to measure methylmercury levels in fish to reflect the actual levels of methylmercury contamination in Malaysia.
